# Development of EEG connectivity from preschool to school-age children

**DOI:** 10.3389/fnins.2023.1277786

**Published:** 2024-01-11

**Authors:** Jiannan Kang, Wenqin Mao, Juanmei Wu, Xinping Huang, Manuel F. Casanova, Estate M. Sokhadze, Xiaoli Li, Xinling Geng

**Affiliations:** ^1^Child Rehabilitation Division, Ningbo Rehabilitation Hospital, Ningbo, China; ^2^Department of Biomedical Sciences, University of South Carolina School of Medicine Greenville Campus, Prisma Health System, Greenville, SC, United States; ^3^Duke University, Durham, NC, United States; ^4^State Key Laboratory of Cognitive Neuroscience and Learning, Beijing Normal University, Beijing, China; ^5^School of Biomedical Engineering, Capital Medical University, Beijing, China

**Keywords:** EEG, functional connectivity, effective connectivity, children, brain development

## Abstract

**Introduction:**

Many studies have collected normative developmental EEG data to better understand brain function in early life and associated changes during both aging and pathology. Higher cognitive functions of the brain do not normally stem from the workings of a single brain region that works but, rather, on the interaction between different brain regions. In this regard studying the connectivity between brain regions is of great importance towards understanding higher cognitive functions and its underlying mechanisms.

**Methods:**

In this study, EEG data of children (*N* = 253; 3-10 years old; 113 females, 140 males) from pre-school to schoolage was collected, and the weighted phase delay index and directed transfer function method was used to find the electrophysiological indicators of both functional connectivity and effective connectivity. A general linear model was built between the indicators and age, and the change trend of electrophysiological indicators analyzed for age.

**Results:**

The results showed an age trend for the functional and effective connectivity of the brain of children.

**Discussion:**

The results are of importance in understanding normative brain development and in defining those conditions that deviate from typical growth trajectories.

## 1 Introduction

Ever since Berger et al. (1961) first demonstrated the age-related changes of EEG in infants, children and adults in 1929, many studies have collected normative developmental EEG data to better understand the boundary between the normal and abnormal brain. Deviation from normal development may have a major impact for our ability as an adult, and may be related to disorders such as ADHD (Kerson et al., 2022), autism (Geng et al., 2023), and schizophrenia (Khare and Bajaj, 2022). Thus, knowledge of developmental trajectories and normal growth of the brain is of great importance when investigating risk factors and treatment options for neuropsychiatric disorders.

Higher cognitive functions are often dependent on the interaction of different brain regions, in this regard studying the connectivity between brain regions is an important step towards understanding higher cognitive functions and underlying pathophysiological mechanisms. Brain connectivity refers to patterns of anatomical connectivity (Wang et al., 2018), statistical dependence (functional connectivity) (Zhang et al., 2021), or causal interactions (effective connectivity) (Chai et al., 2019) between different areas of the nervous system that correspond to individual neurons, groups of neurons, or anatomically isolated brain regions. Connectivity patterns are formed by structural junctions, such as synapses or fibrous pathways, or is represented statistically as causal relationships.

EEG, as a non-invasive acquisition method, is mainly used to measure neural activity signals, with high temporal resolution. EEG studies have found some changes in functional connectivity with development. van Baal et al. (2001) measured children at 5 years of age with a follow up at 7 years of age and reported continuous changes in EEG coherence. In the gradual development of EEG coherence, occasional “growth spurts” were observed which coincide with periods of discontinuous development in cognition. Bell reported that novice crawlers (1–4 weeks) displayed greater coherence than either pre-locomotor infants or experienced crawlers suggesting a dependence between coherence and behavior in 8 months-old infants (Bell and Fox, 1996). In a recent study, Bosch-Bayard et al. (2022) described the maturation of EEG effective connectivity in healthy infants during the first year of life, and they found that age-related connectivity changes were mostly long-range and bilateral.

So far, there is no specific research on the development trend of EEG connectivity into school-age children from 3 to 10 years of age and used the weighted phase lag index (wPLI) and directed transfer function (DTF) method to find the electrophysiological indicators of functional and effective connectivity of typical development children. This allowed us to build a general linear model between the electrophysiological indicators as a function of age. These findings provide important clues for the study of the development pattern of brain connectivity during childhood and lay a foundation for the study of brain development in pathological disorders.

## 2 Methods

### 2.1 Participants

A total of 253 children were enrolled in the present study. Participants included children aged between 3 and 10 years from the kindergarten and school. We provided informed consent to child guardians. The inclusion criteria were as follows:

(1) Children had no mental development disorders (e.g., autism, hyperactivity disorder, developmental retardation), and no family history of other related mental diseases.
(2) Children had no nervous system disease or other serious physical diseases, no history of severe brain injury and high fever and convulsion.
(3) All children were right-handed.

The exclusion criteria were: (1) children with any mental development disorders; (2) left-handed children; (3) children whose parents did not sign informed consent forms.

The present research was conducted according to the Declaration of Helsinki. Nervous system development transpires rapidly during childhood, so we divided our study sample into 7 groups with 1 year of age as the interval. The age information is shown in Table 1.

**Table 1:** Information table of children.

Group	Sex (number)	Age (years old)
	(Male/female)	Mean ± SD
3–4 years old	18/14	3.537 ± 0.236
4–5 years old	21/17	4.483 ± 0.265
5–6 years old	19/12	5.485 ± 0.276
6–7 years old	22/23	6.449 ± 0.321
7–8 years old	13/11	7.578 ± 0.351
8–9 years old	21/18	8.608 ± 0.275
9–10 years old	26/18	9.467 ± 0.241

### 2.2 Data recording

A 128-channel HydroCel Sensor Net System (Electrical Geodesics, Inc.) was used for data recording. All children cleaned their hair and scalp before EEG data collection and EEG was recorded in a shielding room. Participating children were seated on a comfortable chair with eyes open. During the EEG acquisition process, the electrode impedance was controlled below 50 kΩ, the sampling frequency was 1,000 Hz, the reference electrode was Cz, and the acquisition time was not less than 5 min.

### 2.3 Data analysis

#### 2.3.1 EEG data preprocessing

Data preprocessing was based on Matlab R2016a and EEGlab. The processing flow was: (1) EEG data were down sampled to 200 Hz and was band-pass filtered between 1 and 45 Hz; (2) the independent component analysis (ICA) algorithm was used to remove artifacts induced by ocular and muscle movements; (3) choosing and replacing the bad channels by the adjacent channels if the channel impedance was greater than 50 kΩ or the amplitude was more than 200 μV; (4) data was segmented for a period of 10 s. (5) All channels were re-referenced to an average reference and 62 electrodes were used for next analysis.

For the calculation of connectivity in brain regions, five brain regions were selected including frontal region (F), left temporal region (LT), parietal region (P), right temporal region (RT) and occipital region (O). For the calculation of across hemispheric connectivity, eight brain regions were used including left frontal region (LF), right frontal region (RF), left temporal region (LT), left parietal region (LP), right parietal region (RP), right temporal region (RT), left occipital region (LO) and right occipital region (RO).

#### 2.3.2 Functional connectivity

Functional connectivity is a measure of the degree of dependence or correlation between two signals. In this study we used the weighted phase lag index (Vinck et al., 2011) as a robust functional connectivity, because of its relative immunity to volume conduction effects (Lee et al., 2017; Comsa et al., 2019).

We calculated the cross-spectrum of two preprocessed EEG epochs, and the weighted phase lag index (wPLI) value in four canonical frequency bands, that is delta (1–4 Hz), theta (4–8 Hz), alpha (8–13 Hz) and beta (13–30 Hz). In regard to the functional connectivity in one brain region, the wPLI was averaged among all electrode pairs and then averaged among all the epochs. On the other hand, connectivity was averaged among the wPLI values of all the electrode pairs in two different brain regions and then averaged among epochs across hemispheric brain regions.

#### 2.3.3 Effective connectivity

Effective connectivity refers to the degree of causal dependence between two signals, but can also calculate the direction of information flow between the two brain regions. In this study, the DTF algorithm was used based on the Granger (1969) causality.

Due to the cancellation of the directionality of effective connectivity when averaging brain regions, we only calculated effective connectivity across hemispheres. Averaging all channel EEG signals in the left and right frontal regions (LF and RF), left and right temporal regions (LT and RT), left parietal and right parietal regions (LP and RP), and left and right occipital regions (LO and RO) brain regions across hemispheres. Since the multivariate autoregressive (MVAR) model assumes that the signal was stationary, we used data segmentation methods to treat the EEG signal as a quasi-stationary signal. Specifically, the preprocessed 62 channel EEG signal was divided into 10 s intervals (2,000 data points). For each segment of data, calculate the directed transfer function (DTF) within the frequency range of 1–30 Hz, with a frequency step of 1 Hz.

Constructed a surrogate data set by randomly disrupting the phase on each channel of the multichannel data, then DTF_surrogate_ was calculated on this multi-channel surrogate data. Repeated this procedure 100 times and obtained 100 DTF_surrogate_ values. Then the 100 values were arranged from largest to smallest and the fifth DTF_surrogate_ value was set to the threshold (significance level set to 0.05). The DTF value calculated from the EEG signal (DTF_EEG_) was compared with this threshold. If the DTF_EEG_ value was greater than the threshold, it was considered to have a significant causal relationship (He et al., 2011). Finally, all significant DTF_EEG_ values were averaged across all epochs and four canonical frequency bands used as the effective connectivity across hemispheric brain regions.

#### 2.3.4 Statistical analysis

In this study, Shapiro–Wilk was used to test the wPLI and DTF of each brain region in each frequency band. They were both normally distributed, and repeated measurement analysis of variance (ANOVA) was performed for the connectivity of each frequency band: age group (3 years-old group, 4 years-old group, 5 years-old group, 6 years-old group, 7 years-old group, 8 years-old group, 9 years-old group) was used as the inter-test factor, and brain region was used as the intra-test factor to analyze the main effect of the group and the interaction effect between age groups and brain regions. For connectivity in brain regions, the repetitive measurement factors include five levels: frontal region, parietal region, left temporal region, right temporal region and occipital region’ For connectivity across hemispheric brain regions, the repeated measurement factors included 12 levels: left frontal-right temporal region (LF-RT), left frontal-right parietal region (LF-RP), left frontal-right occipital region (LF-RO), left temporal-right frontal region (LT-RF), left temporal-right parietal region (LT-RP), left temporal region and right occipital region (LT-RO), left parietal region-right frontal region (LP-RF), left parietal region-right temporal region (LP-RT), left parietal region-right occipital region (LP-RO), left occipital-right frontal region (LO-RF) Left occipital-right temporal region (LO-RT) and left occipital-right parietal region (LO-RP).

In order to explore the change trend of functional connectivity and effective connectivity with age during brain development, a general linear model (GLM) (shown in Equation 1) was established to analyze the effect of age:


(1) $$Y=\beta_{0}+\beta_{1}\times \mathrm{age}$$

where, *Y* is the dependent variable and age is the independent variable, $\beta_{0}$ represents intercept, $\beta_{1}$ represents the slope, and the change curve of the functional connectivity in brain regions and across hemispheric brain regions as well as effective connectivity across hemispheric brain regions with age are investigated. The significance level is *p* < 0.05.

## 3 Results

### 3.1 Repeated measurement ANOVA of functional connectivity

The results of repeated measurement ANOVA in brain regions and across hemispheric brain regions were presented in Tables 2, 3. Regarding the functional connectivity in brain regions, significant differences were observed for the main effect of age groups in the delta frequency band (*F* = 2.374, *p* = 0.0307), as well as a significant interaction effect between age groups and brain regions (*F* = 1.713, *p* = 0.0182). Similarly, the main effect of age groups was also significant in alpha frequency band (*F* = 16.002, *p* = 0.000), and there was a significant interaction effect as well (*F* = 8.353, *p* = 0.000).

**Table 2:** The results of repeated measurement ANOVA in brain regions and age groups at four frequency bands.

	Brain region (*F*, *p*)	Age group (*F*, *p*)	Brain region × group (*F*, *p*)
Delta	*F* = 4.172; *p* = 0.0024^*^	*F* = 2.374; *p* = 0.0307^*^	*F* = 1.713; *p* = 0.0182^*^
Theta	*F* = 1.894; *p* = 0.1095	*F* = 0.0963; *p* = 0.4512	*F* = 0.700; *p* = 0.8551
Alpha	*F* = 18.198; *p* = 0.0000^*^	*F* = 16.002; *p* = 0.0000^*^	*F* = 8.353; *p* = 0.0000^*^
Beta	*F* = 5.874; *p* = 0.0001^*^	*F* = 0.609; *p* = 0.7229	*F* = 0.842; *p* = 0.6839

^*^Denotes *p* < 0.05.

**Table 3:** The results of repeated measurement ANOVA across hemispheric brain regions and age groups at four frequency bands.

	Brain region (*F*, *p*)	Age group (*F*, *p*)	Brain region × group (*F*, *p*)
Delta	*F* = 1.406; *p* = 0.1630	*F* = 2.627; *p* = 0.0178^*^	*F* = 1.471; *p* = 0.0087^*^
Theta	*F* = 1.377; *p* = 0.1770	*F* = 0.976; *p* = 0.4424	*F* = 0.839; *p* = 0.8191
Alpha	*F* = 1.038; *p* = 0.4097^*^	*F* = 14.366; *p* = 0.0000^*^	*F* = 1.616; *p* = 0.0014^*^
Beta	*F* = 1.959; *p* = 0.0286^*^	*F* = 1.666; *p* = 0.1309	*F* = 0.301; *p* = 0.0540

^*^Denotes *p* < 0.05.

For the functional connectivity across hemispheric brain regions, the main effect of age groups showed significant differences in the delta frequency band (*F* = 2.673, *p* = 0.0178), and the interaction effect between age groups and brain regions was also significant (*F* = 1.471, *p* = 0.0087). Similarly, the main effect of age groups was also significant in the alpha frequency band (*F* = 14.366, *p* = 0.000), along with a significant interaction effect (*F* = 1.616, *p* = 0.0014).

### 3.2 Development trend of functional connectivity in brain regions

**Figure 1:**
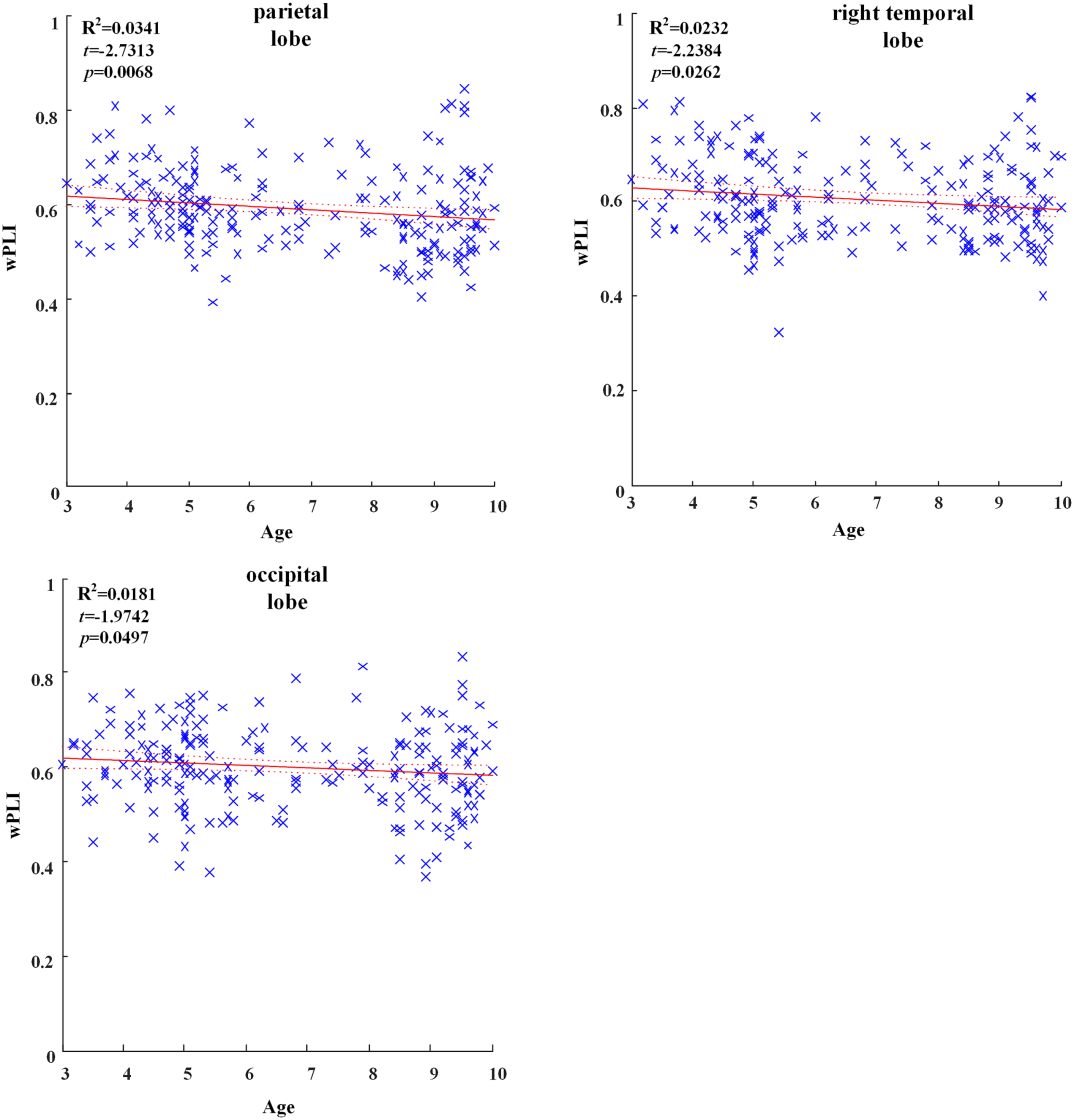
Significant changes of functional connectivity in brain regions in delta frequency band with increasing age.

In all brain regions of delta frequency band, the GLM fitting straight line was calculated and results showed decreasing trend of functional connectivity with age in seven groups, in which parietal region (*t* = −2.7313, *p* = 0.0068), right temporal region (*t* = −2.2384, *p* = 0.0262) and occipital region (*t* = −1.9742, *p* = 0.0497) showed significant decreasing trend, as shown in Figure 1.

**Figure 2:**
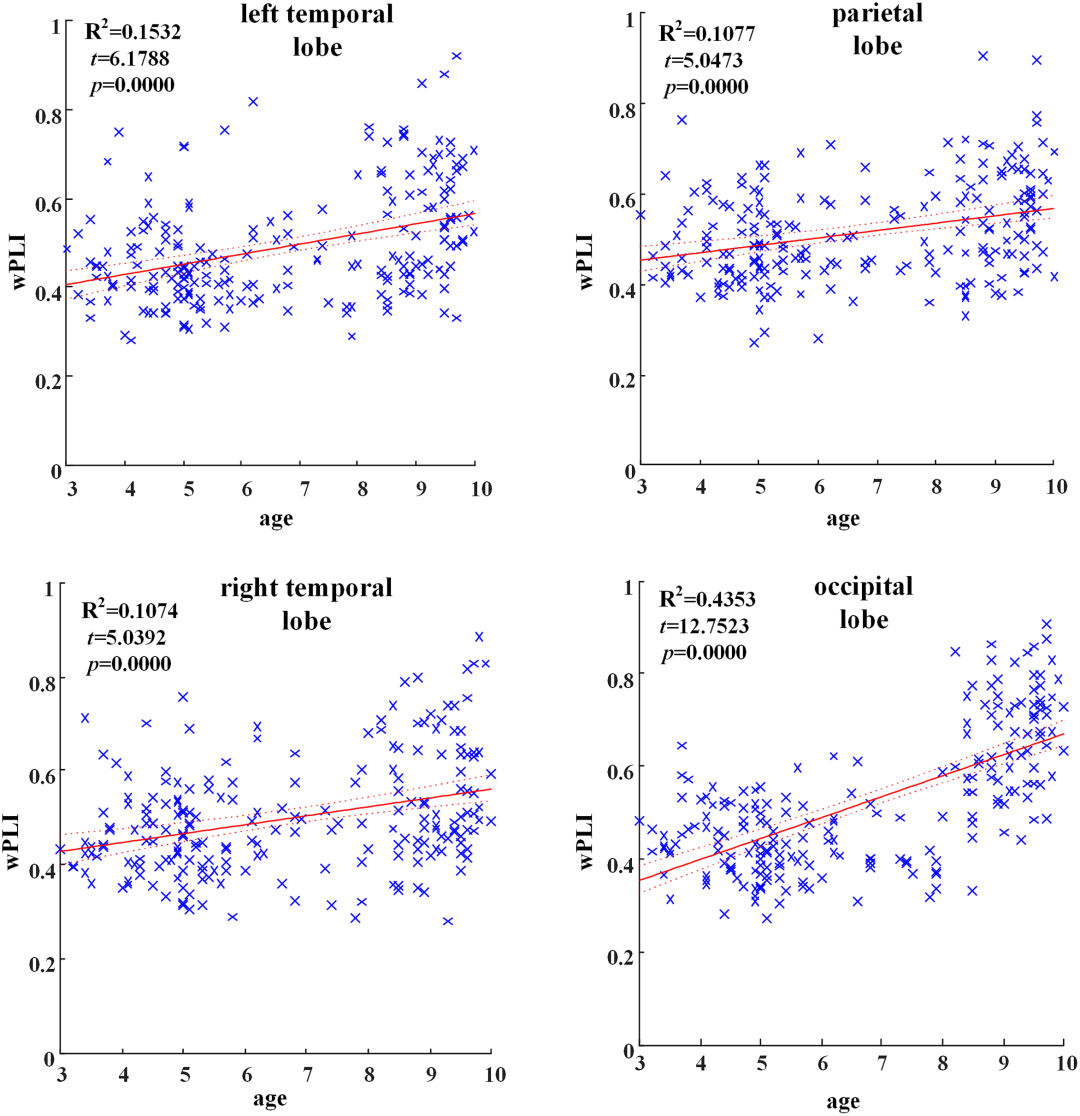
Significant changes of functional connectivity in brain regions in delta frequency band with increasing age.

In all brain regions of alpha frequency band, the GLM fitting straight line was calculated and results showed an increasing trend of functional connectivity with age in seven groups, in which parietal region (*t* = 5.0473, *p* = 0.000), left temporal region (*t* = 6.1788, *p* = 0.000), right temporal region (*t* = 5.0392, *p* = 0.000) and occipital region (*t* = 12.7523, *p* = 0.000) showed significant increasing trend, as shown in Figure 2.

### 3.3 Development trend of functional connectivity across hemispheric brain regions

**Figure 3:**
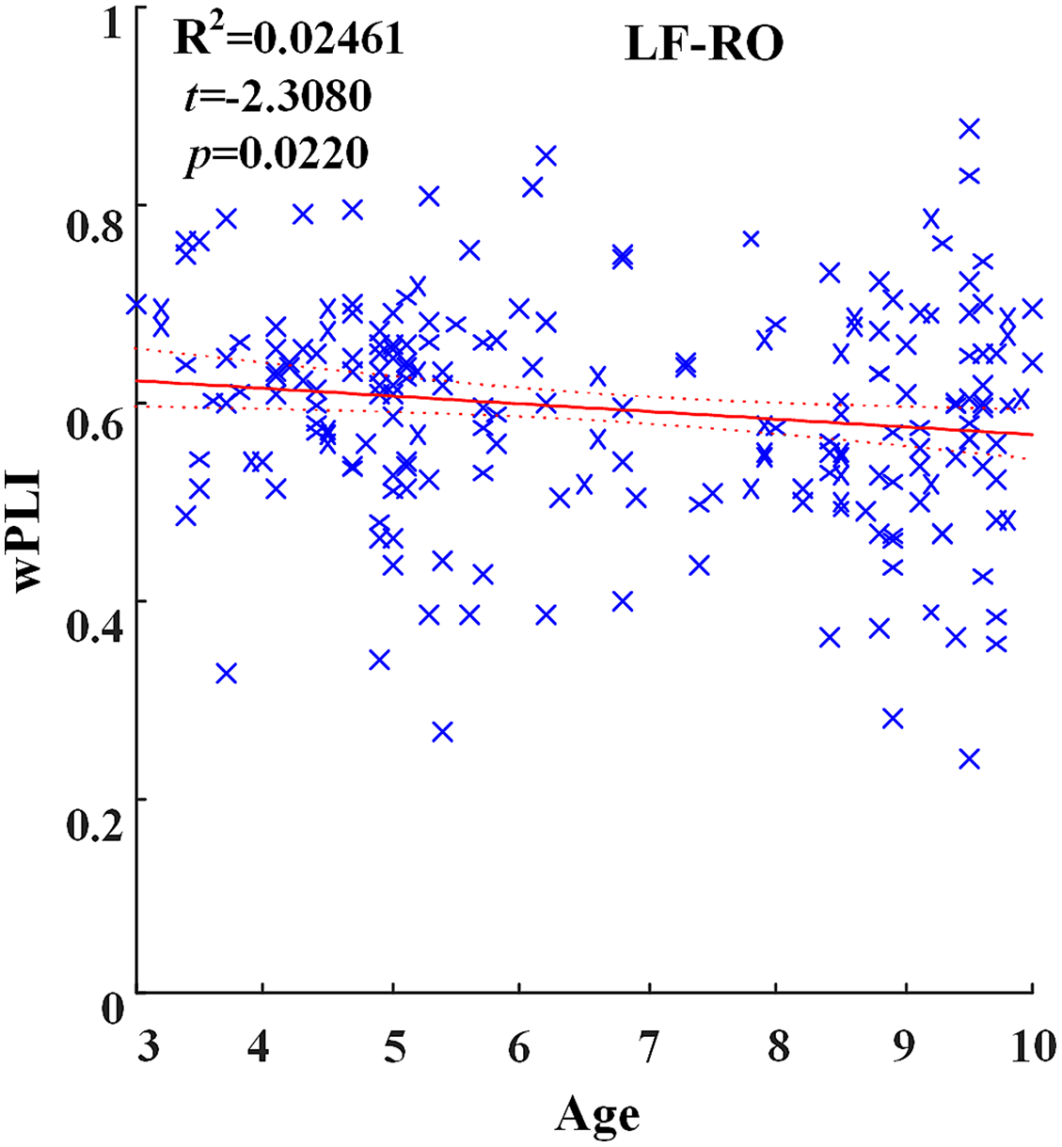
Significant changes of functional connectivity across hemispheric brain regions of delta frequency band with increasing age.

Across hemispheric brain regions of the delta band, the GLM fitting straight line was calculated and results showed a decreasing trend of functional connectivity with age in seven groups, LF-RO (*t* = −2.3080, *p* = 0.0220), as shown in Figure 3.

**Figure 4:**
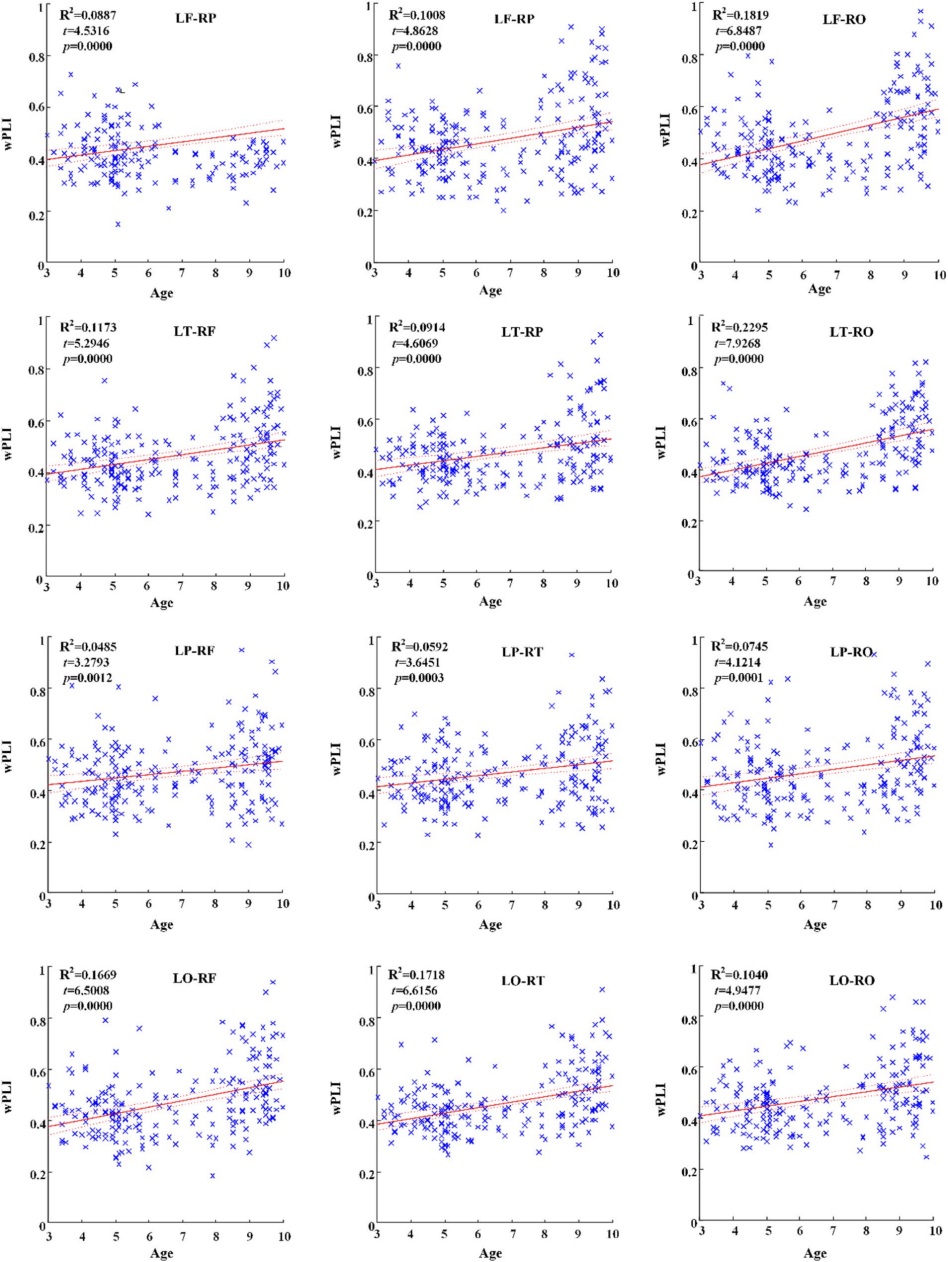
Significant changes of functional connectivity across hemispheric brain regions of alpha band with increasing age.

Across hemispheric brain regions of alpha frequency band, the GLM fitting straight line was calculated and results showed an increasing trend of functional connectivity with age in seven groups, in which LF-RP (*t* = 4.5316, *p* = 0.0000) and many other connectivity across hemispheric brain regions showed significant increasing trend, as shown in Figure 4.

### 3.4 Development trend of effective connectivity across hemispheric brain regions

For the effective connectivity across hemispheric brain regions, the main effect of groups showed significant differences in the alpha frequency band (*F* = 18.821, *p* = 0.000), and the interaction effect between groups and brain regions was also significant (*F* = 2.844, *p* = 0.0000).

**Figure 5:**
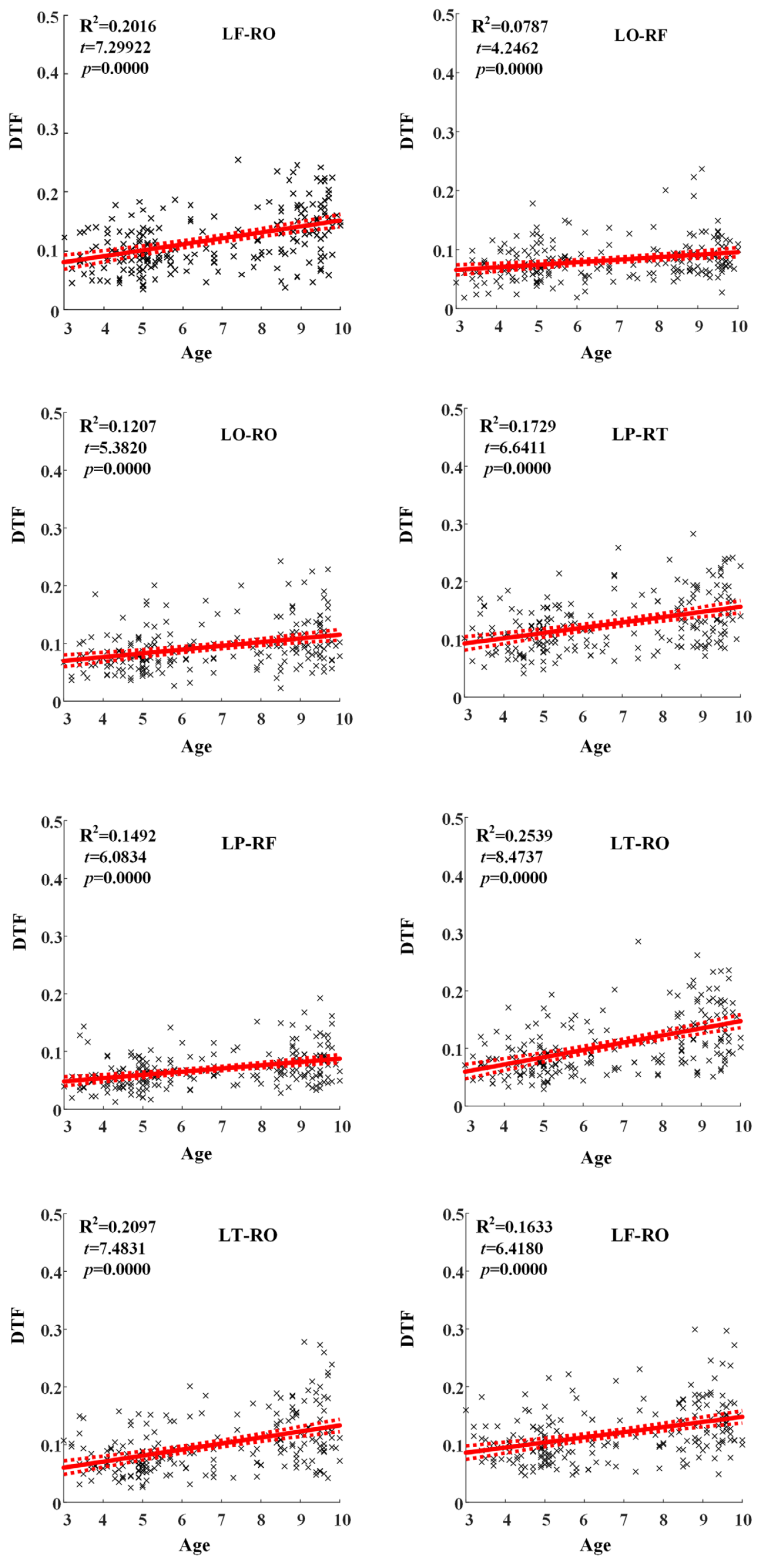
Significant changes of effective connectivity across hemispheric brain regions in alpha frequency band with increasing age.

Across hemispheric brain regions of alpha frequency band, the GLM fitting straight line was calculated and results showed an increasing trend of effective connectivity with age in seven groups, in which from LF to RO (*t* = 7.2992, *p* = 0.0000) and many other connectivity across hemispheric brain regions showed significant increasing trend, as shown in Figure 5.

**Figure 6:**
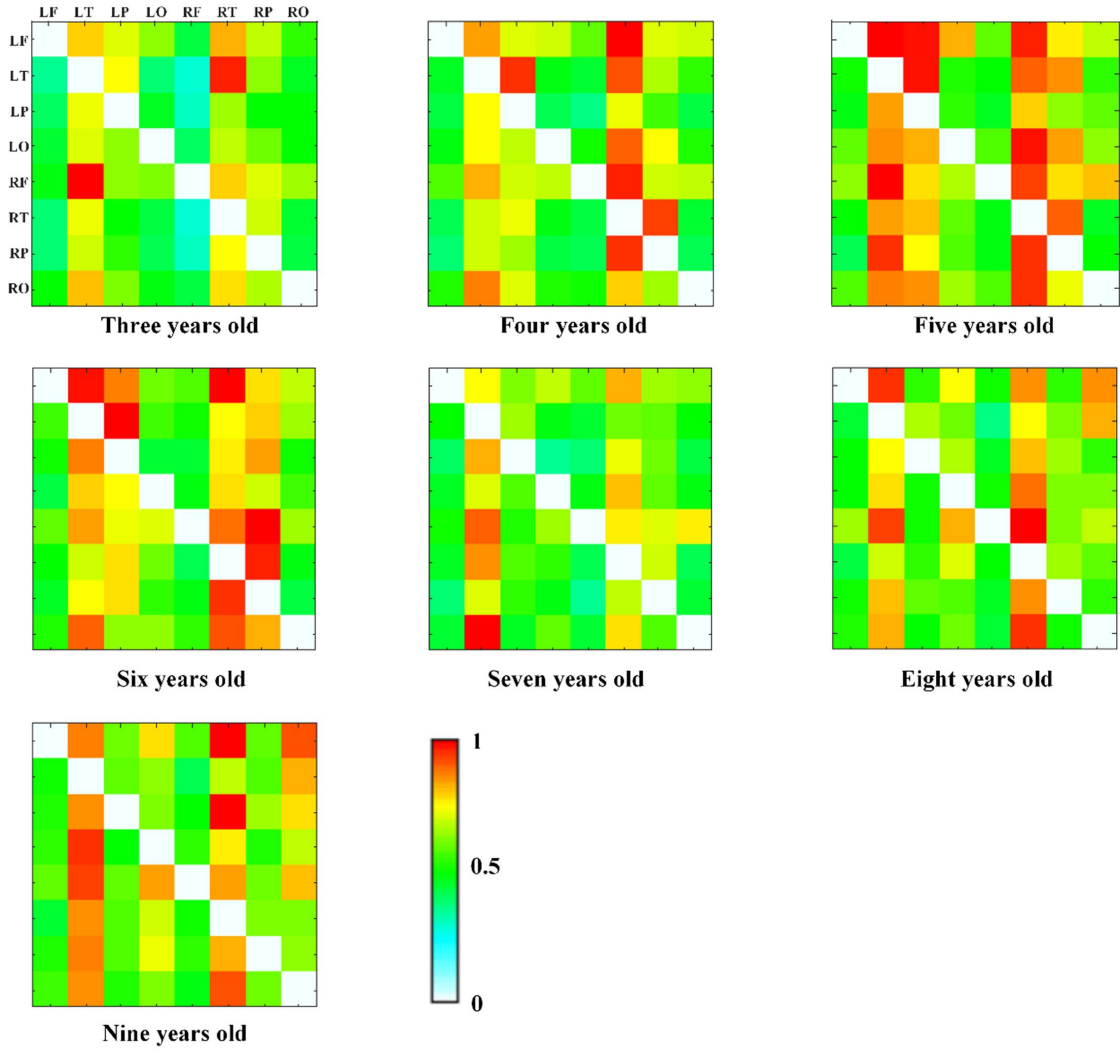
The alpha band DTF effective connectivity matrix across age groups, with normalized DTF matrix values represented by color (red = 1, white = 0).

We also calculated the connectivity matrix of each group in alpha frequency band, as shown in Figure 6, and found that the effective connectivity between the temporal region and other brain regions is more obvious, especially at the age of 5 years old.

## 4 Discussion

In this study, 253 typically developing children 3 to 10 years old were included to explore EEG functional connectivity and effective connectivity to reveal the development trend of brain connectivity. Firstly, the results of the repeated measures ANOVA revealed significant effects of age groups on both functional and effective connectivity patterns in the brain. Significant differences were observed in the delta and alpha frequency bands, both within brain regions and across hemispheric brain regions. These findings emphasize the influence of age on connectivity and suggest potential implications for brain development. More specifically, the GLM model results showed that the brain functional connectivity in all brain regions in delta frequency band decreased with age increasing, while the alpha frequency band increased with age increasing. The functional connectivity across hemispheric brain regions in delta frequency band decreased with age increasing, and alpha frequency band showed a significant upward trend with age increasing. Similarly, the effective connectivity across hemispheric brain regions in alpha frequency band showed an upward trend with the increase of age. Besides, we found that the effective connectivity at 5 years of age was more obvious between temporal region and other brain regions through the connectivity matrix diagram. In general, we found that with the growth of children’s age, the functional connectivity of slow wave band (delta and theta) in specific brain regions gradually decreased, and the alpha band of functional connectivity and effective connectivity gradually increased.

### 4.1 The development of functional connectivity with age

The main potential mechanism of information processing and integration in the brain is caused by the synchronous oscillation of neuron groups. The synchronization phenomenon in EEG is the key to establish the information exchange between different brain regions. Brain development is mainly determined by the formation of myelin sheath and synapse, which can produce more effective interconnection between different brain regions. The increase of synapses means more junctions and better interaction between different structures, which may be reflected in the increased connection value (Womelsdorf et al., 2007). From toddler to late adolescence, the macro pattern of axonal projection in human brain has basically remained unchanged to a large extent, but it also experienced dramatic functional changes, leading to network refinement. These functional changes are mediated by increasing myelin formation, changes in axon diameter and junction density, and changes in neurochemical mediators. Previous studies have confirmed the positive correlation between structure and function, and reported that this relationship increases with age. With aging, the degree of integration of structural binding continues to improve, and the degree of separation continues to decrease (Hagmann et al., 2010).

In this study, we found that the connectivity trend of delta frequency band decreased and alpha frequency band increased with increasing age, both in the brain region and across hemisphere brain region. Previous research results showed that the mature normative pattern of EEG images in children and adolescents involved a decline in total power and absolute power in all bands, especially in slow-wave bands. With development, a substitution process has taken place, in which the slow-wave power has been replaced by faster activities. The increase and decrease of these regional complementarities are mainly from the rear to the front of the region and between regions, which is considered to reflect regional maturity (Whitford et al., 2007). Our findings complement the previous research results and provided more direct proof. The reason for the decrease of delta band connectivity with age may be related to increasing cortical differentiation (Salazar et al., 2021). Huttenlocker (1976) found that in all cortical areas, the density of neurons and synapses reached the maximum value in the first 2 years after birth, and then gradually decreased from the second year to the 16th year. The age of subjects in our study was 3–10 years old, which was within this age range. With the increase of age, the initial brain synapses were overproduced, but with the continuous increase of information received, the synapses were constantly pruned until they became stable. This view also explained why the delta band of young children accounted for a high proportion, and with the continuous pruning of synapses, it gradually decreased.

Research showed that the alpha band neural oscillation was regarded as an active inhibition mechanism, which controlled sensory information processing according to cognitive correlation, and the phase synchronization of alpha oscillation can adjust the brain’s integration function of information (Sadaghiani et al., 2012). Alpha oscillation phase locking across the cortex can integrate separated information in time to promote the integration of multi-sensory motion or more complex perception-action cycle (Palva and Palva, 2007). Marosi et al. (1992) reported that the coherence of the parietal and temporal regions increased with age in the alpha frequency band. With aging, the increase of functional connectivity may be the result of myelin formation in the process, because myelin formation is not completed until around the age of 20 (Brophy and Vance, 1976), our subjects were in the age range of childhood. The increased connectivity of alpha frequency band may also relate to the increased development of coupling in thalamic-cortical projection, because thalamic-reticular nucleus plays a very important role in the generation and synchronization of alpha rhythm (Steriade et al., 1990). Our study thus confirmed the previous findings and further clarified the changes of functional connectivity in children with aging.

### 4.2 The development of effective connectivity with age increasing

Brain development is a mature process spanning the period from childhood to adolescence to adulthood. By the age of 6, the brain volume reaches 95% of the size of adults, which means that most of the volume changes have occurred. The brain is undergoing a key fine-tuning process and the form of cell maturation, such as myelin formation and spinal cord pruning, which allows cognitive development (Petanjek et al., 2008). This development mode is related to the brain maturation theory of slow wave fluctuations in BOLD signals in fMRI (Lüchinger et al., 2012), gray matter density decreasing with age in sMRI research (Chugani, 1998). However, few studies reported the change trend of effective connectivity during these stages of development, especially in childhood. To our limited knowledge, this is the first study using EEG to analyze the changes of brain effective connectivity in children.

Previous studies have confirmed that the precise synchronization of neural oscillations and neuronal discharges support the time coordination of distributed brain processes and was an important criterion for brain functional maturity. The data showed that the improvement of detection rate and the reduction of response time during development were accompanied by the increase of EEG synchronization. In the younger age group, synchronization was mainly found between the frontal and temporal regions and between the two hemispheres (Uhlhaas et al., 2009), and our results were also consistent with it. We found that the effective connectivity showed an upward trend with increasing age, which further supplemented the previous research results. Some studies have also shown that EEG consistency and stable development pattern can lead to a coupling growth period in the cerebral cortex. From 4 to 6 years old, the changes of fronto-occipital coupling, left fronto-temporal coupling and local right fronto-polar pairing were involved in it (Womelsdorf et al., 2007). The results of fMRI showed that those areas that served the main functions (such as motor and sensory systems) may mature earlier, while the advanced areas involved in the integration of these main functions may mature later (Tanaka et al., 2012). The brain regions and connectivity with the most significant age-related nonlinear changes were mainly located in the frontal and temporal cortex. These results were consistent with the most significant age-related changes in the size of neurons in the frontal and temporal regions in the postmortem study (Terry et al., 2022). Our results also showed an effective connectivity enhancement at the age of 5 years, especially in the temporal region in the alpha frequency band. Some studies of ERP had relevant evidence which showed the enhancement of the alpha band synchronization. In a study for children aged 6–11 years, the event-related alpha activity of the auditory task was evaluated. The alpha band response synchronization increased with the age of children, and the response amplitude at the parietal region was greater, which may reflect gradual frontal location with growth (Yordanova et al., 2002). These results showed that different anatomical systems are sequential in the process of cortical development, and the time of EEG changes observed in our study coincided with the time of Piaget’s theory of human cognitive development.

Our results provided important clues for the study of brain connectivity development patterns of children before and after school age, and provide a reference for the future study of brain development of children with brain diseases. However, there were some limitation in our study. Firstly, the connectivity analysis was conducted in the electrode-based brain region without using source location tools. Secondly, the lack of EEG data of younger children and adults means that it is impossible to complete the trend analysis of all age groups. Thirdly, there is no comparison of different connectivity calculation methods.

## Data availability statement

The raw data supporting the conclusions of this article will be made available by the authors, without undue reservation.

## Ethics statement

The studies involving humans were approved by Ethics Committee of Beijing Normal University. The studies were conducted in accordance with the local legislation and institutional requirements. Written informed consent for participation in this study was provided by the participants’ legal guardians/next of kin. Written informed consent was obtained from the minor(s)’ legal guardian/next of kin for the publication of any potentially identifiable images or data included in this article.

## Author contributions

JK: Writing – original draft, Conceptualization, Formal analysis. WM: Data curation, Writing – review & editing. JW: Data curation, Writing – review & editing. XH: Data curation, Writing – review & editing. MC: Writing – review & editing. ES: Writing – review & editing. XL: Formal analysis, Writing – review & editing. XG: Formal analysis, Software, Writing – review & editing.
